# Artificial Intelligence Approaches for Osteoporotic Fracture Risk Prediction Using Administrative Health Data: A Systematic Review

**DOI:** 10.1007/s00223-026-01563-1

**Published:** 2026-06-26

**Authors:** Benjamin Bakke Hansen, Kasper Westphal Leth, Nana Roust Hansen, Bo Abrahamsen, Nicholas Rubek Fuggle, Jan Christian Brønd, Katrine Hass Rubin

**Affiliations:** 1 Research Unit OPEN, Department of Clinical Research, University of Southern Denmark, 5000 Odense, Denmark; 2 OPEN (Open Patient data Explorative Network), Odense University Hospital, 5000 Odense, Denmark; 3 Department of Medicine, Holbæk Hospital, 4300 Holbæk, Denmark; 4 MRC Lifecourse Epidemiology Center, University of Southampton, Southampton, UK

**Keywords:** Artificial intelligence, Machine learning, Fracture risk prediction, Administrative data, Register data, Systematic review

## Abstract

To systematically review Machine Learning (ML) fracture risk prediction models developed solely using administrative data, evaluating their development, performance, risk of bias, and concerns regarding applicability. A systematic search was conducted in PubMed, Embase, IEEE, and Web of Science following PRISMA guidelines (up to November 2025). We included studies developing or validating ML models for osteoporotic fracture risk in adults using administrative data without clinical measurements. Risk of bias and concerns for applicability were assessed using the PROBAST tool. Seven studies were included from 3435 initial records. A range of of ML models were utilized, including Random Forests, XGBoost, LASSO-regularized Logistic Regression and Neural Networks. Discriminative performance was moderate-to-good, with best Area Under the Curve (AUC) values ranging from 0.818 for osteoporotic fractures in general to 0.905 for hip fractures only. While five studies showed low risk of bias, five raised applicability concerns due to using specific subpopulations or features with database characteristics. Only two studies used external validation and none directly evaluated clinical utility of the models. ML models using solely administrative data show some promise as scalable, automated tools for fracture risk prediction without requiring manual clinical input. However, clinical implementation is hindered by limited external validation and a lack of formal utility evaluation of the models. Future research should prioritize more rigorous external validation and recalibration of the existing models, more so than the development of novel models, ensuring they are robust, interpretable, and integrable into future clinical workflows.

**Supplementary Information:**

The online version contains supplementary material available at 10.1007/s00223-026-01563-1.

## Introduction

Osteoporotic fractures represent a growing public health challenge [1]. Osteoporosis is a chronic, progressive disease characterized by low bone mass and structural deterioration of bone tissue, leading to increased bone fragility and susceptibility to fracture [1]. Osteoporotic fractures, especially hip and vertebral fractures, are associated with reduced quality of life, considerable morbidity and mortality, and high utilization of healthcare resources [1–5]. Globally, the number of individuals at high risk of fracture is projected to double between 2010 and 2040 [6]. In Europe alone, the incidence of osteoporotic fractures is expected to reach 5.34 million individuals by 2034 [2]. Despite this substantial burden, a gap persists, where many high-risk individuals remain both unidentified and untreated. Current approaches to fracture risk assessment primarily rely on tools such as the Fracture Risk Assessment Tool (FRAX®), which combines clinical risk factors with or without bone mineral density (BMD) to estimate 10-year fracture probability [7, 8]. While effective, these tools require data such as information on parental fracture history or individual smoking habits that are often not captured accurately in clinical or administrative databases or requirespecific clinical measurements (e.g., DXA scans), limiting their utility for population-wide screening and more automated risk stratification. While FRAX scores can be approximated using some public health database sources [9], in practice FRAX scoring requires manual data entry by the healthcare professional or by the patients themselves. The use of fully routine administrative health data for identifying individuals at high fracture risk has gained increasing attention as a scalable alternative [10, 11]. Because administrative data, such as billing claims and diagnostic codes, are routinely collected, models based on such data can be fully automated and integrated into existing clinical workflows without requiring manual clinician input. However, traditional modelling techniques may struggle with the high dimensionality and sparsity of administrative datasets.

Artificial Intelligence (AI), specifically its subfield Machine Learning (ML), offers potential solutions to these data challenges. ML models, ranging from “simpler” model archetypes like Logistic Regression [12], tree-based ensembles like Random Forests [13] and XGBoost [14] to complex Neural Networks [15] can ingest vast numbers of variables to discover non-linear patterns and complex interactions [16]. While various ML models have been developed using radiographs, CT scans, or electronic health records (EHR) enriched with laboratory values [17–23], these models retain the limitations of requiring specialized clinical data. A potential opportunity lies in leveraging ML models solely built on routinely collected administrative data to create autonomous and low-barrier tools.

To date, no study has systematically summarized the available methods in this specific context. The aim of this systematic review is to examine the current evidence on ML-based fracture risk prediction models developed solely using administrative data. Specifically, the review will identify existing models, evaluate their development and performance and assess their risk of bias and concerns for applicability using the PROBAST tool.

## Methods

This study was conducted in accordance with the Preferred Reporting Items for Systematic Reviews and Meta-Analyses (PRISMA). The protocol was registered in the international prospective register of systematic reviews PROSPERO (Registration No. CRD420251119855) [24].

### Eligibility Criteria

Eligibility criteria were defined according to the PICO framework, as detailed in our registered protocol [24]. We included studies of adults (≥ 18 years) where AI/ML-based prediction models were developed or validated using administrative or registry data. The primary outcome was risk of major osteoporotic fractures (MOF), defined as fractures of hip, clinical spine, humerus, or wrist. Comparative analyses between different ML approaches and their reported performance metrics were eligible. Full details are available in the published protocol.

Inclusion criteria during the screening phases were as follows: (1) participants aged ≥ 18 years; (2) use of MLapproaches to predict fracture risk; (3) reporting at least one measure for model performance.

Exclusion criteria during the screening phases were: (1) studies analysing only risk factors without developing specific ML models predicting fracture risk; (2) ML models utilizing data on non-administrative measurement results (e.g., blood tests, BMD, X-rays, cognitive status); (3) studies using solely classical statistical approaches like logistic or Cox regression; (4) meta-analyses, reviews, case reports, editorials, letters, conference material, protocols, or notes.

### Definitions

In this review, we examined prediction models that estimated the risk of MOF or hip fractures.

We define ML as an approach to developing prediction models where performance is reported, and model evaluation is based on cross-validation or a fixed division into training and validation sets. Thus, it is not the underlying model type that defines ML in the strictest sense, but the methodological approach to model development in combination with using one or more algorithms for prediction modelling. We further restricted our focus to models based on administrative data that can be applied directly by clinicians without requiring additional information from new clinical measurements or self-reported patient data.

In this review, administrative data are defined as information routinely available in health registries or electronic health databases. These data are generated as part of the billing or operation of healthcare systems and include a variety of coded information on diagnoses, procedures, healthcare contacts, and medication use. They are stored electronically and can be automatically extracted at the coding level (e.g., ICD diagnostic codes, procedure codes, and ATC codes for medications), without the need for manual chart review. This definition excludes data that require new clinical assessments, self-reported data, or additional diagnostic procedures (e.g., lifestyle information, clinical measurements, laboratory tests, or imaging data).

### Search Strategy

Relevant studies were retrieved from PubMed, Embase, The Institute of Electrical and Electronics Engineers (IEEE), and Web of Science, all searched on August 12, 2025, with a 10-year lookback period. To ensure inclusion of the most recent evidence, an updated search was conducted on November 06, 2025. A search string was developed using a combination of MeSH terms and free-text keywords related to osteoporotic fractures and MOF types, ML and AI approaches, and predictive modelling, as detailed in Supplementary Material A. The individual search string results for each database are also provided in PROSPERO [24].

### Screening, Data Extraction, and Reporting

Two researchers (KWL and NRH) independently screened the titles and abstracts of all retrieved articles. Discordances were discussed and resolved with the help of a third author (JCB). Based on the predefined inclusion and exclusion criteria, full-text articles were reviewed, and reasons for exclusion were documented. Data extraction from the included studies was independently performed by two researchers (NRH and BBH) using the Checklist for Critical Appraisal and Data Extraction for Systematic Reviews of Prediction Modelling Studies (CHARMS) checklist [25], ensuring consistent and comprehensive extraction of key study characteristics, methods, and results (Tables 1 and 2). Extraction was further supplemented with study-specific items such as feature lookback periods, fracture prediction interval, and fracture outcome. See the full list of extracted items in Supplementary Tables S1 and S2.

**Table 1:** Main feature categories used in prediction models per included study

Feature category	Sub-category of feature	Almog et al [27]	Engels et al [28]	Khalid et al [33]	Li et al [29]	Möller et al [31]	Reinold et al [30]	Rietz et al [32]
Model name		Crystal Bone	SVLFG	IFRISK	CDARS	FREM_Ver2_	GePaRD	FREM_ML_
Demographics	Age	**Index**	**Index**	**Index**	**Index**	**Index**	**Index**	**Index**
	Sex	**Index**	**Index**	**Index**	**Index**	**Index**	**Index**	**Index**
Diagnoses & morbidities	All available diagnosis codes	**2 years**			**1 year**	**15 years**		**15 years**
	Specific pre-selected diagnoses		**2 years**	Complete PH*			Complete PH*	
	Proxied by medication		**7 months**					**15 years**
	Comorbidity indices (CCI)			Complete PH*		**15 years**		**15 years**
Medications & prescriptions	All available prescription codes				**1 year**	**15 years**		**15 years**
	Specific pre-selected medications		**7 months**	**1 year**			**1 year**	
	Polypharmacy (drug count)						**1 year**	**15 years**
Fracture history	Prior fracture status	**2 years**	**2 years**	**Complete PH**	**1 year**	**15 years**	*Exclusion criteria*	**15 years**
Healthcare utilization	Number of diagnoses/visits	**2 years**						**15 years**
	Nursing home residency		*Exclusion criteria*				Complete PH*	
Proxies for lifestyle or clinical factors	Alcohol use						Complete PH*	**15 years**
	Smoking						Complete PH*	
	Obesity/BMI						Complete PH*	**15 years**

Displays the main feature categories and sub-categories identified in the included studies. The text in bold within each cell show the lookback period used for that feature category. Italic cells indicate that the feature was used as an exclusion criterion rather than a model feature, which is colored blue. Abbreviations: Complete *PH* Complete Patient History available prior index

**Table 2:** Best test or validation performance achieved by each prediction model per fracture outcome and sex

Study	Sample sizes (N)	Test dataset type	Age (years)	Sex	Fracture outcome	Fracture rate	Imbalance handling	Model type	AUC (95% CI)	Sensitivity	Specificity
*Osteoporotic fracture models*											
Almog et al (Crystal Bone)	3,408,494 (Train set) 192,590 (Test set)	Internal hold-out	50 +	Both	Any Osteoporotic Fx	6.5%	Oversampling of fracture events	Ensemble	0.818	0.693	0.777
Rietz et al (FREM_ML_)	1,462,650 (Train set) 487,790 (Test set)	Internal hold-out	45 +	Both	Major Osteoporotic Fx	0.90%	Positive/Negative class weighting	DART Boosting (LightGBM)	0.77 (0.76;0.77)	0.69	0.71
	718,919 (Train set) 236,637 (Test set)			Women		1.3%			0.75 (0.73;0.76)	0.63	0.76
	748,161 (Train set) 247,985 (Test set)			Men		0.60%			0.73 (0.72;0.74)	0.62	0.72
Möller et al (Frem_Ver2_)	308,050 (Train set) 102,683 (Cut-off Validation set)	Internal hold-out	65–69	Men	Major Osteoporotic Fx	0.5%	None	Logistic Regression (LASSO)	0.72 (0.70;0.74)	0.79	0.51
	439,024 (Train set) 146,341 (Cut-off Validation set)		45–64	Women		0.6%			0.69 (0.68;0.71)	0.81	0.41
Khalid et al (IFRISK)	33,567 (Train set) 92,262 (Indep. Validation set)	External validation (CRPD)	50 +	Women	Major Osteoporotic Fx	NR	None	Logistic Regression (LASSO)	0.66 (0.65;0.67)	NR	NR
	7,834 (Train set) 23,634 (Indep. Validation set)			Men					0.66 (0.64;0.69)		
	33,567 (Train set) 92,262 (Indep. Validation set)			Women	Any Fx Outcome				0.64 (0.63;0.65)		
Reinold et al (GePaRD)	430,218 (Train set) 107,554 (Test set)	Internal hold-out	75–84	Men	Any Fx requiring hospitalization	1.26%	None	Logistic Regression (Model H)	0.63	0.59	0.62
	525,009 (Train st) 131,252 (Test set)			Women		2.50%		Random Forest (Model G)	0.60	0.60	0.54
Khalid et al (IFRISK)	7,834 (Train set) 23,634 (Indep. Validation set)	External validation (CRPD)	50 +	Men	Any Fx Outcome	NR	None	Logistic Regression (LASSO)	0.58 (0.48;0.68)	NR	NR
*Hip fracture models*											
Li et al (CDARS)	55,300 (Train set) 1,008 (Independent Validation)	External validation	60 +	Men	Hip Fracture	3.60%	None	Neural Network	0.905 (0.86;0.95)	0.806	0.823
Rietz et al (FREM_ML_)	1,462,650 (Train set) 487,790 (Test set)	Internal hold-out	45 +	Both	Hip Fracture	0.24%	Positive/Negative class weighting	DART Boosting (LightGBM)	0.85 (0.85;0.86)	NR	NR
Li et al (CDARS)	73,541 (Train set) 2,038 (Independent Validation)	External validation	60 +	Women	Hip Fracture	7.11%	None	Gradient Boosting Machine	0.845 (0.81;0.88)	0.724	0.802
Möller et al (Frem_Ver2_)	308,050 (Train set) 102,683 (Cut-off Validation set)	Internal hold-out	65–69	Men	Hip Fracture	0.5%	None	Logistic Regression (LASSO)	0.77 (0.74;0.79)	0.88	0.50
	439,024 (Train set) 146,341 (Cut-off Validation set)		45–64	Women		< 0.1%			0.76 (0.70;0.82)	0.31	0.95
Khalid et al (IFRISK)	33,567 (Train set) 92,262 (Indep. Validation set (CPRD))	External validation (CRPD)	50 +	Women	Hip Fracture	NR	None	Logistic Regression (LASSO)	0.73 (0.72;0.75)	NR	NR
	7,834 (Train set) 23,634 (Indep. Validation set (CPRD))			Men					0.72 (0.69;0.75)	NR	NR
Engels et al (SVLFG)	230,469 (Train set) 57,618 (Test set)	Internal hold-out	65 +	Both	Hip Fracture	2.70%	Random Undersampling	Logistic Regression (Forward Selection)	0.704	NR	NR

Reports the best performing models sorted by AUC metric on the test or validation datasets from each included study and stratified first by prediction outcome; ie. models that predict osteoporotic fractures in general or solely hip fractures. Then further stratified by sex to allow representation of multiple models from studies that split their models by sex. Abbreviations: *Fx* Fracture, *LASSO* Least absolute shrinkage and selection operator, *GBM* Gradient boosting machine, *DART* Deep adaptive resampling technique

### Quality Assessment

The risk of bias and applicability of the included studies were assessed independently by two reviewers (NRH and BBH) using the Prediction Model Risk of Bias Assessment Tool (PROBAST) [26], which evaluates four domains (participants, predictors, outcomes, and analysis) for risk of bias and three (participants, predictors, outcome) for concerns regarding applicability. Disagreements at any stage were resolved by discussion or, if necessary, by consultation with a third author (JCB).

## Results

**Fig. 1:**
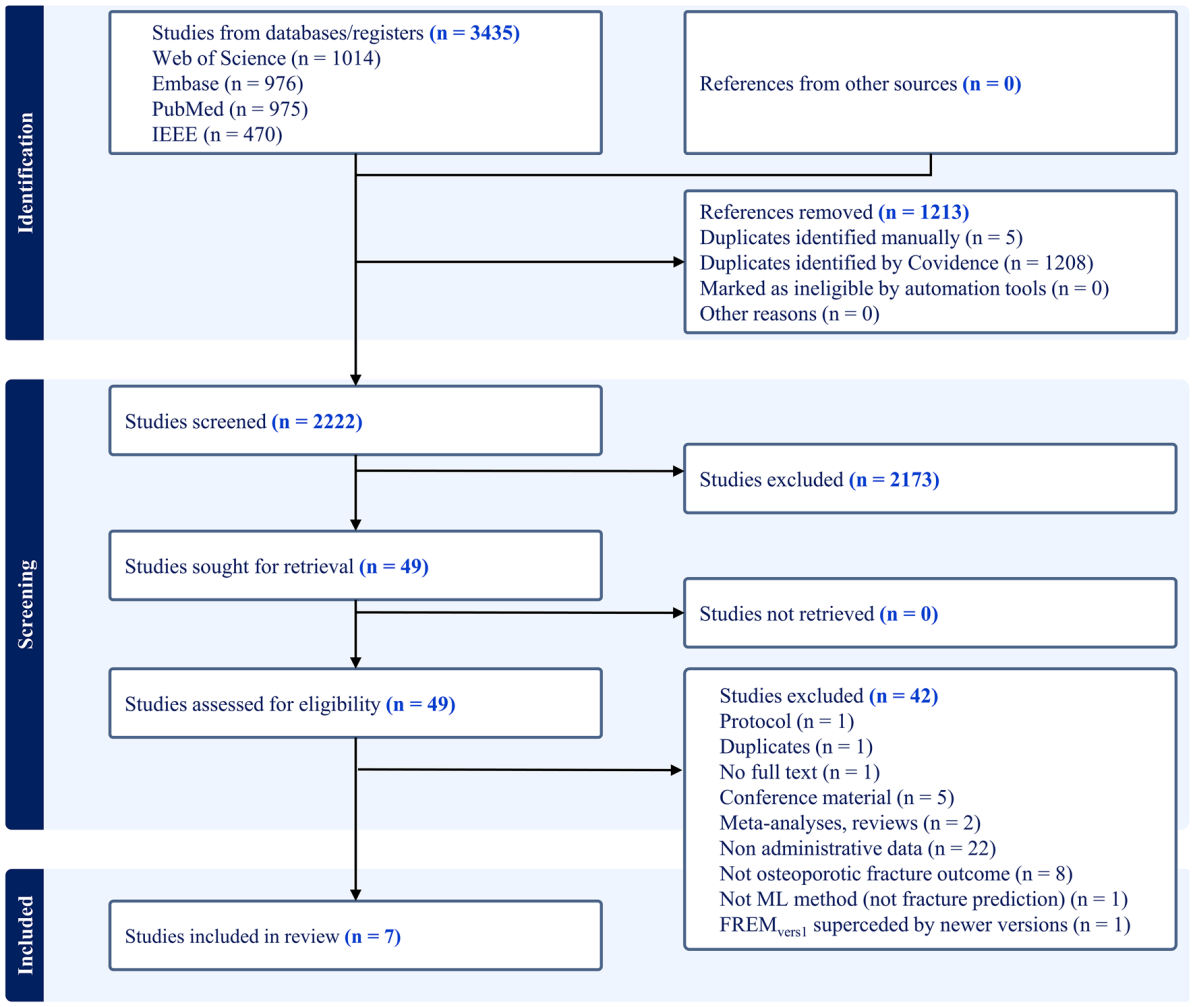
PRISMA flow chart of the review process and included studies. PRISMA flow diagram of the study selection process. The figure illustrates the identification, screening, eligibility and inclusion phases. A total of 3435 records were identified from the four databases. After removing duplicates and applying exclusion criteria, 7 studies were included in the final review. Abbreviations: PRISMA = Preferred Reporting Items for Systematic Reviews and Meta-analyses; ML = Machine Learning; FREM = Fracture Risk Evaluation Model

A total of 3435 studies were retrieved from the databases, including 975 from PubMed, 976 from Embase, 470 from IEEE, and 1014 from Web of Science. Of these, 1213 were removed as duplicates. A total of 2222 articles underwent title and abstract screening, and 49 articles were assessed in full text. Following full-text review, 7 articles were included [27–33]. The review process is shown in Fig. 1.

FREM_Ver1_ [34] did meet all eligibility requirements, but was excluded because FREM_Ver2_ [31] and FREM_ML_ [32] are the most recent models from the research group of FREM (Fracture Risk Evaluation Model), which are direct model expansions of FREM_Ver1_ making FREM_Ver1_ redundant for reporting.

### Characteristics of Included Studies

The overall characteristics of the seven included studies are presented in Table S1. All studies were published between 2020 and 2025 using data sources from the United States [27], Germany [28, 30], Hong Kong [29], Spain [33], United Kingdom [33] and Denmark [31–33] (Table S1). Four of the studies named their prediction models: the Fracture Risk Evaluation Model (FREM) in two studies; FREM_ver2_ [31], and FREM_ML_ [31, 32], the Imminent Fracture Risk Prediction Tool (IFRISK) [33], and the Crystal Bone Model [27]. For this review model names were given to the remaining studies based on their database acronyms: SVLFG Model [28], GePaRD Model [30] and the CDARS Model [29] (Table S1). The studies utilized large-scale administrative data sources, including administrative EHRs, administrative claims data, and national registry data. The various sample sizes used to train the models were substantial, ranging from 41,401 in IFRISK (OBP cohort) [33] to over 2.4 million in the study in the FREM models [31, 32] (Table S1).

All studies included models for both men and women, either combined or stratified by sex, with participants’ ages ranging from 45 years or older in the FREM models [31, 32] to 65 years or older in SVLFG and GePaRD [28, 30].

All included models were developed to predict future fracture risk over intervals ranging from 1 year in FREM, Crystal Bone and IFRISK [27, 31–33] to 10 years in CDARS [29], using feature lookback periods ranging from 7 months in SVLFG [28] to 15 years in the FREM models [31, 32] (Table S1). The primary fracture outcomes predicted were MOFs or hip fractures alone, however, the GePaRD model also predicted any fracture leading to hospitalization, and IFRISK predicted any fracture excluding those of the face, skull and digits [30, 33] (Table S1).

A variety of ML model types were developed, ranging from ‘simpler’ models like LASSO regularized Logistic Regression, to Random Forests, eXtreme Gradient Boosting (XGBoost), Light Gradient-Boosting Machine (LightGBM), Long Short-Term Memory (LSTM) and neural networks (Table S1). These methods range from systems inspired by the brain to find patterns, such as Neural Networks and LSTMs, to Ensemble Models like XGBoost, Random Forests and LightGBM that essentially use the “wisdom of the crowd” by combining many simple models to vote on a final answer [35]. All studies performed internal validation using a holdout set, where the CDARS model [29] additionally underwent external validation on an independent cohort and IFRISK conducted double external validation using datasets from Denmark and UK [33].

### Features Included in the Prediction Models

The prediction models incorporated a wide array of feature categories directly extracted or derived from administrative data (Table 1). All seven studies included the features of age and sex. Each study utilized some combination of features for information on “Diagnoses & Morbidities”, “Medications & Prescriptions” and “Healthcare Utilization”. All studies except the GePaRD Model included “Fracture History” as features in the prediction models, where they instead chose to use it as an exclusion criterion [30]. The SVLFG study was also unique in choosing to exclude patients with a history of nursing home residency. Features for”Proxies for lifestyle or clinical factors” were also used in the FREM_ML_ model [32] and the GePaRD Model [30].

### Model Performances

The performance of the prediction models is sorted by the metric AUC and varied by fracture outcome, age, sex, and model type (Tables 2 and S2).

For the prediction of osteoporotic fractures, the ensemble model of Crystal Bone [27], which included both men and women aged 50 and older, achieved the highest AUC of 0.818, while demonstrating a sensitivity of 0.693 and a specificity of 0.777 on the internal holdout set (Tables 2 and S2). IFRISK was the only externally validated osteoporotic fracture model, which had lower AUC performances of around 0.66 and crucially did not report sensitivity or specificity from their models.

For the prediction of hip fractures, models generally demonstrated higher discriminatory abilities as evident by higher overall AUC performance metrics. The CDARS model, a Neural Network modelled by Li et al. [29] for men aged 60 and older, achieved an AUC of 0.905 in an independent external validation cohort, alongside a sensitivity of 0.806 and a specificity of 0.823. In the same study, a Gradient Boosting Machine yielded an AUC of 0.845 for women, which was comparable to the FREM_ML_ [32] sex-combined hip fracture model evaluated on internal hold-out (AUC 0.85) (Tables 2 and S2).

### Risk of Bias and Concern for Applicability Assessment

**Fig. 2:**
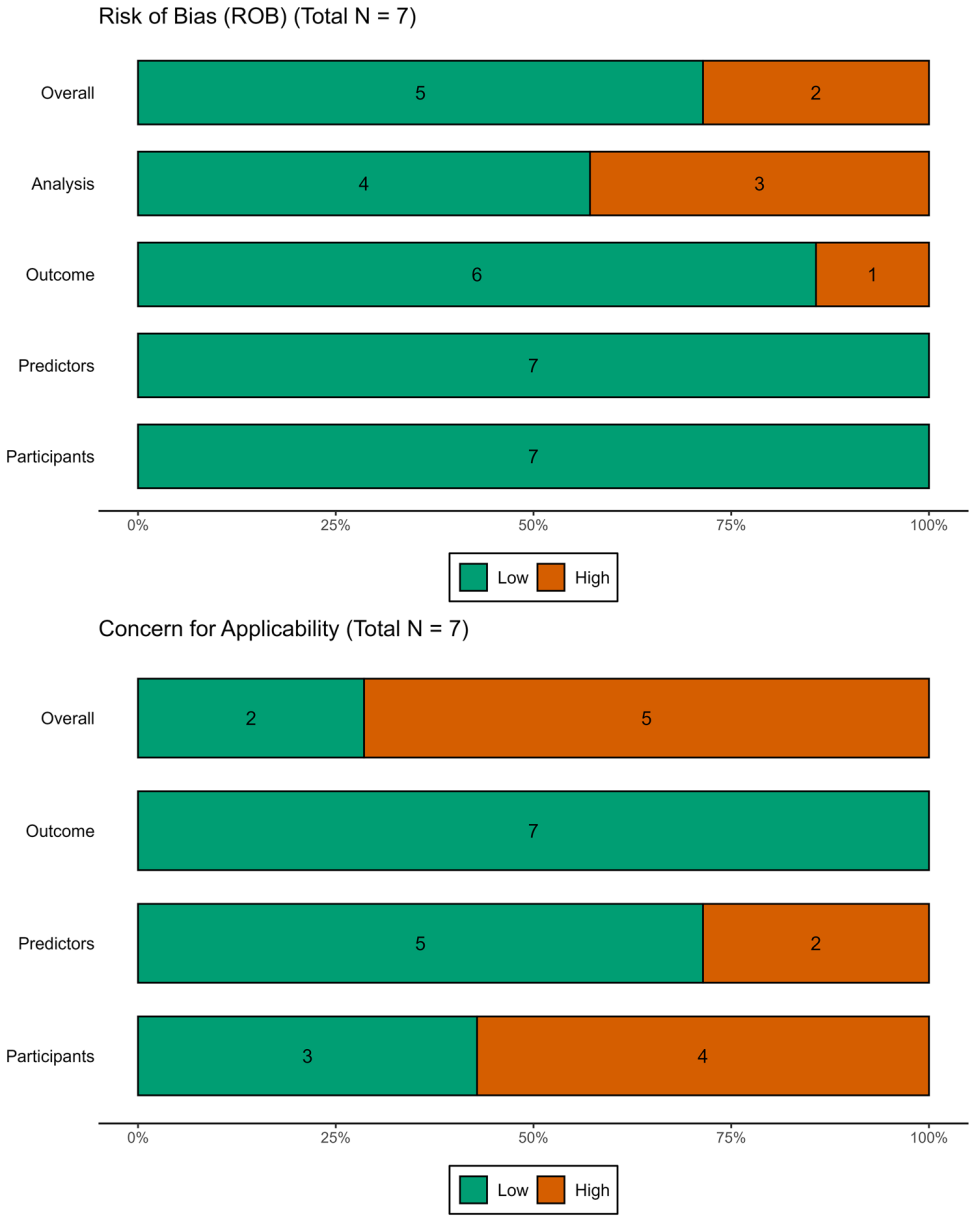
Risk of bias and concern for applicability by PROBAST criteria. Bar graphs visualizing the score evaluations for Risk of Bias and Concern for Applicability as pertaining to the PROBAST criteria. Abbreviations: PROBAST = Prediction Model Risk of Bias Assessment

The risk of bias and concern for applicability of the included studies were assessed using the PROBAST criteria (Table S3 and Fig. 2).

Overall, the risk of bias was evaluated as low for five out of seven studies, with some concern regarding the use of random undersampling [36] to balance the fracture rates in each class in the SVLFG model by Engels et al. [28]. IFRISK [33] also included over 80 candidate features, but had total fracture events in the low hundreds in their entire training dataset, thereby not adhering to a commonly accepted heuristic requirement of at least 10 events per variable to prevent unstable model beta-coefficients in logistic regression models [37].

Five studies had high concern regarding the applicability of the models [27–29, 33]. In the development of the Crystal Bone Model [27], an EHR database was used and not a purely administrative database in the strictest sense, leading to concerns regarding the generalizability for prediction models in the context of solely administrative data. Similar concerns apply for the CDARS model [29] which also used an EHR database which may be more granular or differently structured than a typical purely administrative database. The CDARS model [29] was developed using a Hong Kong population which provides a very valuable resource, but raises concerns about direct generalizability to other populations. For the IFRISK model [33] the best performing models were developed on individuals with recent initiation of oral bisphosphonates. While this is a highly relevant study population, it raises concern for the generalizability of such models for the broader population. Lastly, the SVLFG Model [28] used data from the German Agricultural Sickness Fund, which is limited to people working in agriculture and their families. This quite specific rural subpopulation raises concerns about how well the models developed on this group generalize to a broader population. Finally, the GePaRD model [30] chose to exclude individuals with prior fracture, which was done because the paper aimed to estimate risk of first fracture specifically. While this is a logical decision for the paper, it leads to concerns for applicability from the perspective of this review, because prior fracture is a very well known and strong predictor of current fracture risk [38].

The remaining included models FREM_ver2_ and FREM_ML_ [31, 32] were found to have low risk of bias and low concern for applicability by the PROBAST guidelines and in the context of the aim of this systematic review. A detailed rationale behind each PROBAST evaluation for each paper, can be found in Supplementary Material B.

## Discussion

### Main Results

The initial database search retrieved 3435 studies, of which 2222 remained after duplicate removal and underwent title and abstract screening. A total of 49 articles were assessed in full text, and ultimately, seven studies met all inclusion criteria and were included in this review. Despite a large body of literature, few models rely solely on administrative data.

The included models [27–33] demonstrated test or validation AUC performance between IFRISK’s 0.58 model for men and Crystal Bone’s AUC of 0.818 for both sexes, when predicting any osteoporotic fracture [27, 33]. The model AUC performances for hip fractures ranged from SVLFG’s 0.704 for both sexes and CDARS’ AUC of 0.905 for hip fracture prediction in men (Table 2) [28, 29]. There was a general tendency towards better model performance when predicting just hip fractures, compared to the more broad categorizations of osteoporotic fractures. While CDARS shows the most promising performance, it is worth noting that CDARS was the only included study to utilize a 10-year prediction horizon [29]. This much longer observation period, compared to the 1–4 year horizons of the other included studies, likely contributed to the higher performance metrics by capturing a greater number of fracture events over time. Short-term prediction windows may inherently be detrimental to the model’s performance metrics, yet we believe estimating the imminent risk of fracture is likely of higher clinical importance, as a high short-term fracture risk strongly signals the necessity for immediate anti-osteoporotic treatment. While these models successfully leveraged administrative data to predict fracture risk, their clinical implementation is currently hindered by limited external validation, clear plans for implementation into clinical workflows and a lack of decision-analytic assessment. Future progress necessitates a concerted effort toward externally validating models across geographically and clinically heterogeneous populations (i.e., diverse ages, sexes and ethnicities). This provides stronger evidence towards the predictive performance being truly robust and equitable. It is well-established that performance metrics on internal validation datasets are often overly optimistic and can degrade substantially when externally validated [39, 40]. Furthermore, models must also be calibrated prior to clinical deployment. While calibration is a monotonic transformation of predicted rankings, meaning it neither improves nor decreases model performance, it ensures that predicted probabilities align with observed outcome frequencies [41]. This is vital for clinical deployment, where predicted probabilities of fracture must accurately reflect the real-world risk within that population.

### Generalizability and Validation

The applicability of a prediction model depends heavily on the diversity of its training data [42]. While the included studies utilized large sample sizes, generalizability remains a concern. The SVLFG model [28] relied on a German agricultural cohort, potentially introducing a selection bias by occupation. Similarly, the IFRISK model [33] focused on high-risk sub-populations of recent fracture or new bisphosphonate users, limiting their utility in a broader primary care setting, and GePaRD excludes patients with prior fracture, making it less than ideal for population-wide fracture risk prediction [30].

Rigorous external validation is required to ensure these models perform well outside their training cohorts [43]. While most studies employed internal hold-out validation or cross-validation, only the CDARS and IFRISK models [29, 33] underwent external validation on independent datasets. The lack of external validation in the remaining studies represents an evaluation gap in performance, as performance metrics often decrease when models are applied to new populations, even if the original models are methodologically sound in development.

### Data Imbalance

Osteoporotic fractures are rare events, incidence rates in the included studies ranged from < 0.1 to 7.11% [29, 31]. Severe class imbalance can bias models towards the majority class of non-fracturing individuals [36]. Approaches to mitigate this varied in the included studies; Crystal Bone [27] used oversampling (SMOTE) [44], FREM_ML_ [32] used class re-weighting, and SVLFG used random undersampling—though the latter likely discarded valuable data, which is a known issue with random undersampling [36]. Notably, four models (FREM_Ver2_, CDARS, IFRISK, GePaRD) did not report any handling of imbalance [29–31, 33]. While the necessity of correcting imbalance is a debated topic, PROBAST guidelines emphasize that it should at least be explicitly addressed [26].

### Clinical Utility

Because fractures are rare events, even models with high AUCs inevitably generate a high volume of false positives. For instance, Reinold et al. reported that applying their GePaRD model to a theoretical population yielded a PPV of only 2–3% [30]. Similarly, Crystal Bone had a precision of only 0.19 despite a good AUC metric [27]. This highlights that AUC can be a misleading metric in imbalanced datasets as it is heavily influenced by the large number of true negatives (i.e., no fractures). Metrics like Area Under the Precision-Recall Curve (AUC-PR) are generally more informative for capturing performance on the minority class in imbalanced datasets [45]. Unlike AUC, which tends to be optimistic in the presence of high amounts of true negatives, AUC-PR focuses strictly on the relationship between precision and recall. This provides a more reliable measure of model performance for the clinical minority class of fracturing patients. However, this metric was not reported enough for the included models to be compared for this review, leading us to rely on AUC as the primary performance metric when ranking model performance.

This implies there is a feasibility issue where models, despite good AUC metrics, are likely unsuitable as diagnostic or decision-making tools for initiating osteoporotic pharmaceutical treatment, as the number of patients unnecessarily treated would be unacceptably high. However, the models do remain potentially feasible as opportunistic screening tools. If the models are used to triage patients for a low-harm intervention, like a DXA scan referral, then the low PPV is more clinically acceptable from a treatment perspective, but unfortunately introduces another immediate challenge. As noted by Li et al., DXA availability is already at maximum capacity and insufficient in many regions [29]. A massive influx of referrals could easily overwhelm diagnostic capacities. Therefore, clinical implementation likely requires dynamic thresholds, allowing healthcare systems to adjust the risk cut-off based on local resource capacity, as suggested and attempted in FREM_Ver2_ by Möller et al. [31]. Future studies must take this dilemma into account before any model is capable of truly becoming beneficial for the patient or the clinician. One example of this could be by a Decision Curve Analysis or net benefit. These metrics are required to determine whether implementing a model actually yields better patient outcomes compared to default strategies such as “treat all” or “treat none” [46]. None of the included studies evaluated clinical utility by this metric, which is a crucial step in terms of evaluating the clinical utility of these models.

### Translating Fracture Risk Models into Clinical Practice

For the models to be trusted by clinicians, they must be interpretable [47]. The FREM_ML_ study was unique in applying Shapley Additive exPlanations (SHAP) values [48] to quantify individual feature contributions. Providing clinicians with transparent explanations, specifically why a patient was flagged as being at risk of fracture, may mitigate trust issues and support informed shared decision-making.

This trade-off between performance and transparency is highlighted by the two best performing models in this review. Crystal Bone utilized advanced natural language processing (NLP) techniques to analyze longitudinal patient histories, achieving an AUC of 0.81 for osteoporotic fracture risk prediction [27]. However, the authors explicitly acknowledged that their deep learning approach remains a “black box” lacking significant interpretability which they deem as the greatest limitation of the model [27]. While they attempted to visualize patient clusters to explain predictions, the model does not provide the clear feature weightings clinicians might require for proper transparency of the underlying model.

Conversely CDARS [29] demonstrated a well known phenomenon, namely that complexity is not necessarily a prerequisite for high or better performance [35]. While their best model was a Neural Network with AUC 0.905, in their validation they found that conventional, and highly interpretable Logistic Regression models actually performed comparably (AUC: 0.898) to the more complex ML models [29]. Conversely, FREM_ML_ [32] utilizing DART Boosting algorithms only marginally outperformed previous iterations of FREM [31, 34] that relied on fewer features and Logistic Regression with LASSO regularization. By utilizing a more interpretable approach, such as Logistic Regression, Li et al. were able to identify and report specific predictors, such as drug classes and comorbidities, potentially facilitating easier integration and acceptance into clinical workflow without the “black box” dilemma [29]. Such interpretability not only fosters clinician trust but also eases the technical integration into clinical workflows, where clear justification for risk stratification is often a prerequisite for successful adoption by both clinician and patient. It could therefore be argued that especially in the face of minor or marginal improvements, one should opt for the simpler and more transparent model [47], and would also align with previous scientific appeals for the principles of parsimonious modelling [49, 50].

### Strengths and Limitations

This systematic review has several methodological strengths. The study adhered to a pre-specified protocol registered in PROSPERO and followed PRISMA guidelines and the PICO framework, ensuring transparency, reproducibility, and a structured approach to study selection. Data extraction was guided by the CHARMS checklist, and two reviewers independently screened titles, abstracts, and full texts, with a third reviewer resolving any discrepancies. The search strategy included multiple major databases, increasing the likelihood of capturing all relevant studies. Another key strength of this review is its specific focus on ML models developed solely using administrative health data. By focusing on these models, we provide insights into tools that are inherently easier to scale and integrate into routine clinical workflows, without requiring manual data entry or additional interventions for the patient. Furthermore, the review highlights the emerging use of interpretability methods, such as SHAP values, which are essential for mitigating clinician distrust in “black box” models.

The review is also subject to limitations. The small number of eligible models and the substantial heterogeneity in study populations, feature sets and outcome definitions, precluded the possibility of a meta-analysis. Direct performance comparisons between studies must therefore be interpreted with some caution. Furthermore, while the use of administrative data could enable high scalability, we must also recognize that such data are typically collected for billing purposes and not research [51]. Contrary or inaccurate evidence can easily arise when comparing findings from such databases across countries and time periods, as local coding practices, healthcare policies and diagnostic criteria heavily influence the underlying data structure and thus any models derived from such data [52]. Therefore, any performance metrics from an automated model that relies on automatically extracted administrative data, must be weighed against the risk of significant discrepancies between this data and clinical reality.

Despite these limitations, this review provides a comprehensive overview of current ML-based fracture risk prediction using administrative data and highlights methodological considerations and evidence that can inform future research and potential clinical implementation of models in this context.

## Conclusion

This systematic review identified seven machine learning models developed solely using administrative data for predicting osteoporotic fracture risk. These models relied on routinely collected information such as diagnoses, medications, healthcare utilization, and fracture history, and applied diverse machine learning techniques, including Random Forests, XGBoost, LASSO regularization, ensemble methods, and Neural Networks. Overall, model performance by AUC was moderate to good, and five out of seven studies were assessed as having a low risk of bias using the PROBAST criteria. Among these, the FREM models demonstrated both low risk of bias and low concern regarding applicability.

Generalizability remains limited, as most models were trained on specific populations and rarely underwent external validation. The two externally validated models did not achieve a low risk of bias and applicability by PROBAST criteria. In addition, performance reporting was often incomplete, with limited reporting of more useful metrics in imbalanced data like AUC-PR. They also lack calibration metrics, interpretability methods, implementation scoping and a proper assessment of the actual clinical utility of the models. This is in part due to the models being in a relatively early experimental stage, but also because no clinically driven and minimally viable performance metrics are currently defined for fracture prediction models. It is also unclear if such models should be used to drive DXA referral, treatment intervention or a even a tertiary clinical goal.

To facilitate better translation into clinical practice, future research should not solely focus on just the development of novel models, but instead prioritize rigorous external validation of existing models identified in this review. Determining whether these already developed models are robust across diverse populations and healthcare systems is a critical step towards establishing their viability for deployment in clinical practice. Consequently, future resources should be more directed toward setting clinically relevant minimum thresholds for model performance, reporting appropriate metrics for imbalanced datasets like AUC-PR, model recalibration, handling and reporting of class imbalances, and ensuring that models remain parsimonious, interpretable, and readily integrable into healthcare workflows.

## Supplementary Information

Below is the link to the electronic supplementary material.

Supplementary file1 (PDF 972 KB) PICO search strings for each database

Supplementary file2 (DOCX 26 KB) PROBAST Grading Rationales

Supplementary file3 (XLSX 14 KB)

Supplementary file4 (XLSX 21 KB)

Supplementary file5 (XLSX 12 KB)

## References

[1] Compston JE, McClung MR, Leslie WD (2019) Osteoporosis. Lancet 393:364–376. 10.1016/s0140-6736(18)32112-3 30696576 10.1016/S0140-6736(18)32112-3

[2] Kanis JA, Norton N, Harvey NC, Jacobson T, Johansson H, Lorentzon M, McCloskey EV et al (2021) SCOPE 2021: a new scorecard for osteoporosis in Europe. Arch Osteoporos 16:82. 10.1007/s11657-020-00871-9 34080059 10.1007/s11657-020-00871-9 PMC8172408

[3] Akkawi I, Zmerly H (2018) Osteoporosis: current concepts. Joints 6:122–127. 10.1055/s-0038-1660790 30051110 10.1055/s-0038-1660790 PMC6059859

[4] Sopina L, Hitz MF, Thygesen LC, Langdahl B, Ladefoged BT, Kruse M (2025) Healthcare and productivity cost of osteoporosis: a Danish register-based quasi-experimental study. Osteoporos IntDOI. 10.1007/s00198-025-07453-w 10.1007/s00198-025-07453-w PMC12089169 40111480

[5] Hernlund E, Svedbom A, Ivergård M, Compston J, Cooper C, Stenmark J, McCloskey EV et al (2013) Osteoporosis in the European Union: medical management, epidemiology and economic burden. Arch Osteoporos 8:136. 10.1007/s11657-013-0136-1 24113837 10.1007/s11657-013-0136-1 PMC3880487

[6] Oden A, McCloskey EV, Kanis JA, Harvey NC, Johansson H (2015) Burden of high fracture probability worldwide: secular increases 2010–2040. Osteoporos Int 26:2243–2248. 10.1007/s00198-015-3154-6 26018089 10.1007/s00198-015-3154-6

[7] Kanis JA (2007) Assessment of osteoporosis at the primary health-care level. Technical report. In: World health organization collaborating centre for metabolic bone diseases, University of Sheffield Medical School, UK

[8] Kanis JA, Harvey NC, Cooper C, Johansson H, Odén A, McCloskey EV (2016) A systematic review of intervention thresholds based on FRAX : a report prepared for the national osteoporosis guideline group and the international osteoporosis foundation. Arch Osteoporos 11:25. 10.1007/s11657-016-0278-z 27465509 10.1007/s11657-016-0278-z PMC4978487

[9] Yang S, Leslie WD, Morin SN, Lix LM (2019) Administrative healthcare data applied to fracture risk assessment. Osteoporos Int 30:565–571. 10.1007/s00198-018-4780-6 30554259 10.1007/s00198-018-4780-6

[10] Curtis EM, Reginster JY, Al-Daghri N, Biver E, Brandi ML, Cavalier E, Hadji P et al (2022) Management of patients at very high risk of osteoporotic fractures through sequential treatments. Aging Clin Exp ResDOI. 10.1007/s40520-022-02100-4 10.1007/s40520-022-02100-4 PMC9076733 35332506

[11] Gupta A, Maslen C, Vindlacheruvu M, Abel RL, Bhattacharya P, Bromiley PA, Clark EM et al (2022) Digital health interventions for osteoporosis and post-fragility fracture care. Ther Adv Musculoskelet Dis 14:1759720x221083523. 10.1177/1759720x221083523 35368375 10.1177/1759720X221083523 PMC8966117

[12] Cox DR (1958) The regression analysis of binary sequences. journal of the royal statistical society. Series B (Methodological) 20:215–242

[13] Breiman L (2001) Random forests. Mach Learn 45:5–32. 10.1023/A:1010933404324

[14] Chen T, Guestrin C (2016) XGBoost: a scalable tree boosting system

[15] Ekundayo OS, Ezugwu AE (2025) Deep learning: historical overview from inception to actualization, models, applications and future trends. Appl Soft Comput 181:113378. 10.1016/j.asoc.2025.113378

[16] Turing AM (1950) I.: computing machinery and intelligence. Mind LIX:433–460. 10.1093/mind/LIX.236.433

[17] Yosibash Z, Trabelsi N, Buchnik I, Myers KW, Salai M, Eshed I, Barash Y et al (2023) Hip fracture risk assessment in elderly and diabetic patients: combining autonomous finite element analysis and machine learning. J Bone Miner Res 38:876–886. 10.1002/jbmr.4805 36970838 10.1002/jbmr.4805

[18] Villamor E, Monserrat C, Del Rio L, Romero-Martin JA, Ruperez MJ (2020) Prediction of osteoporotic hip fracture in postmenopausal women through patient-specific FE analyses and machine learning. Comput Methods Progr Biomed 193:105484. 10.1016/j.cmpb.2020.105484 10.1016/j.cmpb.2020.105484 32278980

[19] de Vries BCS, Hegeman JH, Nijmeijer W, Geerdink J, Seifert C, Groothuis-Oudshoorn CGM (2021) Comparing three machine learning approaches to design a risk assessment tool for future fractures: predicting a subsequent major osteoporotic fracture in fracture patients with osteopenia and osteoporosis. Osteoporos Int 32:437–449. 10.1007/s00198-020-05735-z 33415373 10.1007/s00198-020-05735-z

[20] Chen R, Huang Q, Chen L (2022) Development and validation of machine learning models for prediction of fracture risk in patients with elderly-onset rheumatoid arthritis. Int J Gen Med 15:7817–7829. 10.2147/IJGM.S380197 36276661 10.2147/IJGM.S380197 PMC9581722

[21] Ciuşdel CF, Vizitiu A, Moldoveanu FD, Suciu C, Itu LM (2017) Towards real time machine learning based estimation of fracture risk in osteoporosis patients. In: 2017 international conference on optimization of electrical and electronic equipment (OPTIM) & 2017 Intl aegean conference on electrical machines and power electronics (ACEMP), p 1145–1151

[22] Wu Y, Chao J, Bao M, Zhang N (2023) Predictive value of machine learning on fracture risk in osteoporosis: a systematic review and meta-analysis. BMJ Open 13:e071430. 10.1136/bmjopen-2022-071430 38070927 10.1136/bmjopen-2022-071430 PMC10728980

[23] Smets J, Shevroja E, Hügle T, Leslie WD, Hans D (2021) Machine learning solutions for osteoporosis-a review. J Bone Miner Res 36:833–851. 10.1002/jbmr.4292 33751686 10.1002/jbmr.4292

[24] Hansen BB, Brønd JC, Leth KW, Rubin KH (2025) Review protocol: artificial intelligence for osteoporotic fracture risk prediction using administrative data: a systematic review and meta-analysis. In: PROSPERO 2025 CRD420251119855 10.1007/s00223-026-01563-1 PMC13303480 42350824

[25] Palazon-Bru A, Martin-Perez F, Mares-Garcia E, Beneyto-Ripoll C, Gil-Guillen VF, Perez-Sempere A, Carbonell-Torregrosa MA (2020) A general presentation on how to carry out a CHARMS analysis for prognostic multivariate models. Stat Med 39:3207–3225. 10.1002/sim.8660 32583899 10.1002/sim.8660

[26] Wolff RF, Moons KGM, Riley RD, Whiting PF, Westwood M, Collins GS, Reitsma JB et al (2019) PROBAST: a tool to assess the risk of bias and applicability of prediction model studies. Ann Intern Med 170:51–58. 10.7326/M18-1376 30596875 10.7326/M18-1376

[27] Almog YA, Rai A, Zhang P, Moulaison A, Powell R, Mishra A, Weinberg K et al (2020) Deep learning with electronic health records for short-term fracture risk identification: crystal bone algorithm development and validation. J Med Internet Res 22:e22550. 10.2196/22550 32956069 10.2196/22550 PMC7600029

[28] Engels A, Reber KC, Lindlbauer I, Rapp K, Büchele G, Klenk J, Meid A et al (2020) Osteoporotic hip fracture prediction from risk factors available in administrative claims data: a machine learning approach. PLoS ONE 15(5):e0232969. 10.1371/journal.pone.0232969 32428007 10.1371/journal.pone.0232969 PMC7237034

[29] Li GH, Cheung CL, Tan KC, Kung AW, Kwok TC, Lau WC, Wong JS et al (2023) Development and validation of sex-specific hip fracture prediction models using electronic health records: a retrospective, population-based cohort study. EClinicalMedicine 58:101876. 10.1016/j.eclinm.2023.101876 36896245 10.1016/j.eclinm.2023.101876 PMC9989633

[30] Reinold J, Braitmaier M, Riedel O, Haug U (2022) Potential of health insurance claims data to predict fractures in older adults: a prospective cohort study. Clin Epidemiol 14:1111–1122. 10.2147/CLEP.S379002 36237823 10.2147/CLEP.S379002 PMC9552670

[31] Moller S, Rietz M, Petersen FL, Brond JC, Skjodt MK, Sondergaard J, Abrahamsen B et al (2025) An enhanced fracture risk evaluation model (FREM) using national health data on morbidity and medications. J Bone Miner ResDOI. 10.1093/jbmr/zjaf156 10.1093/jbmr/zjaf156 PMC12765684 41172146

[32] Rietz M, Brond JC, Moller S, Sondergaard J, Abrahamsen B, Rubin KH (2025) Introducing FREM(ML): a decision-support approach for automated identification of individuals at high imminent fracture risk. Arch Osteoporos 20:140. 10.1007/s11657-025-01613-5 41191170 10.1007/s11657-025-01613-5 PMC12589358

[33] Khalid S, Pineda-Moncusi M, El-Hussein L, Delmestri A, Ernst M, Smith C, Libanati C et al (2021) Predicting imminent fractures in patients with a recent fracture or starting oral bisphosphonate therapy: development and international validation of prognostic models. J Bone Miner Res 36:2162–2176. 10.1002/jbmr.4414 34342378 10.1002/jbmr.4414

[34] Rubin KH, Moller S, Holmberg T, Bliddal M, Sondergaard J, Abrahamsen B (2018) A new fracture risk assessment tool (FREM) based on public health registries. J Bone Miner Res 33:1967–1979. 10.1002/jbmr.3528 29924428 10.1002/jbmr.3528

[35] Shwartz-Ziv R, Armon A (2022) Tabular data: deep learning is not all you need. Inf Fusion 81:84–90. 10.1016/j.inffus.2021.11.011

[36] He H, Garcia EA (2009) Learning from imbalanced data. Knowledge and data engineering. IEEE Trans 21(9):1263

[37] Peduzzi P, Concato J, Kemper E, Holford TR, Feinstein AR (1996) A simulation study of the number of events per variable in logistic regression analysis. J Clin Epidemiol 49:1373–1379. 10.1016/s0895-4356(96)00236-3 8970487 10.1016/s0895-4356(96)00236-3

[38] Kanis JA, Johnell O, De Laet C, Johansson H, Oden A, Delmas P, Eisman J et al (2004) A meta-analysis of previous fracture and subsequent fracture risk. Bone 35:375–382. 10.1016/j.bone.2004.03.024 15268886 10.1016/j.bone.2004.03.024

[39] D’Amour A, Heller K, Moldovan D, Adlam B, Alipanahi B, Beutel A, Chen C et al (2022) Underspecification presents challenges for credibility in modern machine learning. J Mach Learn Res 23(Article 226):1–61

[40] Geirhos R, Jacobsen J-H, Michaelis C, Zemel R, Brendel W, Bethge M, Wichmann FA (2020) Shortcut learning in deep neural networks. Nat Mach Intell 2:665–673. 10.1038/s42256-020-00257-z

[41] Niculescu-Mizil A, Caruana R (2005) Predicting good probabilities with supervised learning

[42] Bernhard S, John P, Thomas H (2007) Analysis of representations for domain adaptation. In: Advances in neural information processing systems 19: proceedings of the 2006 conference. MIT Press, p 137–144

[43] Collins GS, Moons KGM, Dhiman P, Riley RD, Beam AL, Van Calster B, Ghassemi M et al (2024) TRIPOD+AI statement: updated guidance for reporting clinical prediction models that use regression or machine learning methods. BMJ 385:e078378. 10.1136/bmj-2023-078378 38626948 10.1136/bmj-2023-078378 PMC11019967

[44] Chawla NV, Bowyer KW, Hall LO, Kegelmeyer WP (2002) SMOTE: synthetic minority over-sampling technique. J Artif Intell Res. 10.1613/jair.953

[45] Saito T, Rehmsmeier M (2015) The precision-recall plot is more informative than the ROC plot when evaluating binary classifiers on imbalanced datasets. PLoS ONE 10:e0118432. 10.1371/journal.pone.0118432 25738806 10.1371/journal.pone.0118432 PMC4349800

[46] Vickers AJ, Elkin EB (2006) Decision curve analysis: a novel method for evaluating prediction models. Med Decis Making 26:565–574. 10.1177/0272989X06295361 17099194 10.1177/0272989X06295361 PMC2577036

[47] Rudin C (2019) Stop explaining black box machine learning models for high stakes decisions and use interpretable models instead. Nat Mach Intell 1:206–215. 10.1038/s42256-019-0048-x 35603010 10.1038/s42256-019-0048-x PMC9122117

[48] Lundberg SM, Erion GG, Lee SI (2018) Consistent individualized feature attribution for tree ensembles. ArXivarXiv:1802.03888

[49] Christodoulou E, Ma J, Collins GS, Steyerberg EW, Verbakel JY, Van Calster B (2019) A systematic review shows no performance benefit of machine learning over logistic regression for clinical prediction models. J Clin Epidemiol 110:12–22. 10.1016/j.jclinepi.2019.02.004 30763612 10.1016/j.jclinepi.2019.02.004

[50] Hand D (2006) Classifier technology and the illusion of progress. Stat Sci 21:1–14. 10.1214/08834230600000006017906740

[51] Hersh WR, Weiner MG, Embi PJ, Logan JR, Payne PR, Bernstam EV, Lehmann HP et al (2013) Caveats for the use of operational electronic health record data in comparative effectiveness research. Med Care 51:S30-37. 10.1097/MLR.0b013e31829b1dbd 23774517 10.1097/MLR.0b013e31829b1dbd PMC3748381

[52] Weiskopf NG, Weng C (2013) Methods and dimensions of electronic health record data quality assessment: enabling reuse for clinical research. J Am Med Inform Assoc 20:144–151. 10.1136/amiajnl-2011-000681 22733976 10.1136/amiajnl-2011-000681 PMC3555312

