# Supplementary material for: Artificial Intelligence Approaches for Osteoporotic Fracture Risk Prediction Using Administrative Health Data: A Systematic Review
Source: Calcif Tissue Int. 2026 Jun 26;117(1):103. doi: 10.1007/s00223-026-01563-1 (PMC13303480; doi:10.1007/s00223-026-01563-1)
Supplement: Supplementary file 1 — Supplementary file1 (PDF 972 KB) PICO search strings for each database [file 223_2026_1563_MOESM1_ESM.pdf]

Artificial Intelligence Approaches for Osteoporotic Fracture Risk Prediction Using Administrative Health Data: A Systematic Review
Calcified Tissue International

Corresponding Author:
Benjamin Bakke Hansen

Supplementary Material B
Search date: 06-11-2025

Pubmed

("Osteoporotic Fractures"[Mesh] OR "Hip Fractures"[Mesh] OR "Spinal Fractures"[Mesh] OR "Humeral Fractures"[Mesh] OR "Radius Fractures"[Mesh] OR "Wrist Fractures"[Mesh] OR (fracture*[Mesh]) OR osteoporotic fracture*[tiab] OR major osteoporotic fracture*[tiab] OR MOF[tiab] OR hip fracture*[tiab] OR spine fracture*[tiab] OR vertebral fracture*[tiab] OR humerus fracture*[tiab] OR humeral fracture*[tiab] OR wrist fracture*[tiab] OR forearm fracture*[tiab] OR radius fracture*[tiab])

AND

("Machine Learning"[Mesh] OR "Artificial Intelligence"[Mesh] OR "Deep Learning"[Mesh] OR "Neural Networks, Computer"[Mesh] OR "Support Vector Machine"[Mesh] OR machine learning[tiab] OR transfer learning[tiab] OR deep learning[tiab] OR artificial intelligence[tiab] OR random forest*[tiab] OR artificial neural network*[tiab] OR ANN[tiab] OR support vector machine*[tiab] OR SVM[tiab] OR gradient boosting*[tiab] OR GBM[tiab] OR Nomogram*[tiab] OR XGBoost[tiab] OR LightGBM[tiab] OR decision tree*[tiab] OR LASSO*[tiab] OR backward selection[tiab] OR forward selection[tiab] OR stepwise selection[tiab] OR recursive elimination[tiab])

AND

(predict*[tiab] OR prognostic[tiab] OR "predictive model*"[tiab])

AND

(y_10[Filter])

Corresponding Author:
Benjamin Bakke Hansen

Supplementary Material B
Search date: 06-11-2025

Search	Actions	Details	Query	Results	Time
#5	•••	>	Search: ((("Osteoporotic Fractures"[Mesh] OR "Hip Fractures"[Mesh] OR "Spinal Fractures"[Mesh] OR "Humeral Fractures"[Mesh] OR "Radius Fractures"[Mesh] OR "Wrist Fractures"[Mesh] OR (fracture*[Mesh]) OR osteoporotic fracture*[tiab] OR major osteoporotic fracture*[tiab] OR MOF[tiab] OR hip fracture*[tiab] OR spine fracture*[tiab] OR vertebral fracture*[tiab] OR humerus fracture*[tiab] OR humeral fracture*[tiab] OR wrist fracture*[tiab] OR forearm fracture*[tiab] OR radius fracture*[tiab])) AND (("Machine Learning"[Mesh] OR "Artificial Intelligence"[Mesh] OR "Deep Learning"[Mesh] OR "Neural Networks, Computer"[Mesh] OR "Support Vector Machine"[Mesh] OR machine learning[tiab] OR transfer learning[tiab] OR deep learning[tiab] OR artificial intelligence[tiab] OR random forest*[tiab] OR artificial neural network*[tiab] OR ANN[tiab] OR support vector machine*[tiab] OR SVM[tiab] OR gradient boosting*[tiab] OR GBM[tiab] OR Nomogram*[tiab] OR XGBoost[tiab] OR LightGBM[tiab] OR decision tree*[tiab] OR LASSO*[tiab] OR backward selection[tiab] OR forward selection[tiab] OR stepwise selection[tiab] OR recursive elimination[tiab]))) AND ((predict*[tiab] OR prognostic[tiab] OR "predictive model*"[tiab])) Filters: **in the last 10 years** Sort by: **Publication Date**	975	03:44:59
#4	•••	>	Search: ((("Osteoporotic Fractures"[Mesh] OR "Hip Fractures"[Mesh] OR "Spinal Fractures"[Mesh] OR "Humeral Fractures"[Mesh] OR "Radius Fractures"[Mesh] OR "Wrist Fractures"[Mesh] OR (fracture*[Mesh]) OR osteoporotic fracture*[tiab] OR major osteoporotic fracture*[tiab] OR MOF[tiab] OR hip fracture*[tiab] OR spine fracture*[tiab] OR vertebral fracture*[tiab] OR humerus fracture*[tiab] OR humeral fracture*[tiab] OR wrist fracture*[tiab] OR forearm fracture*[tiab] OR radius fracture*[tiab])) AND (("Machine Learning"[Mesh] OR "Artificial Intelligence"[Mesh] OR "Deep Learning"[Mesh] OR "Neural Networks, Computer"[Mesh] OR "Support Vector Machine"[Mesh] OR machine learning[tiab] OR transfer learning[tiab] OR deep learning[tiab] OR artificial intelligence[tiab] OR random forest*[tiab] OR artificial neural network*[tiab] OR ANN[tiab] OR support vector machine*[tiab] OR SVM[tiab] OR gradient boosting*[tiab] OR GBM[tiab] OR Nomogram*[tiab] OR XGBoost[tiab] OR LightGBM[tiab] OR decision tree*[tiab] OR LASSO*[tiab] OR backward selection[tiab] OR forward selection[tiab] OR stepwise selection[tiab] OR recursive elimination[tiab]))) AND ((predict*[tiab] OR prognostic[tiab] OR "predictive model*"[tiab])) Sort by: **Publication Date**	1,043	03:44:36
#3	•••	>	Search: (predict*[tiab] OR prognostic[tiab] OR "predictive model*"[tiab]) Sort by: **Publication Date**	2,719,697	03:44:11
#2	•••	>	Search: ("Machine Learning"[Mesh] OR "Artificial Intelligence"[Mesh] OR "Deep Learning"[Mesh] OR "Neural Networks, Computer"[Mesh] OR "Support Vector Machine"[Mesh] OR machine learning[tiab] OR transfer learning[tiab] OR deep learning[tiab] OR artificial intelligence[tiab] OR random forest*[tiab] OR artificial neural network*[tiab] OR ANN[tiab] OR support vector machine*[tiab] OR SVM[tiab] OR gradient boosting*[tiab] OR GBM[tiab] OR Nomogram*[tiab] OR XGBoost[tiab] OR LightGBM[tiab] OR decision tree*[tiab] OR LASSO*[tiab] OR backward selection[tiab] OR forward selection[tiab] OR stepwise selection[tiab] OR recursive elimination[tiab]) Sort by: **Publication Date**	549,137	03:43:46
#1	•••	>	Search: ("Osteoporotic Fractures"[Mesh] OR "Hip Fractures"[Mesh] OR "Spinal Fractures"[Mesh] OR "Humeral Fractures"[Mesh] OR "Radius Fractures"[Mesh] OR "Wrist Fractures"[Mesh] OR (fracture*[Mesh]) OR osteoporotic fracture*[tiab] OR major osteoporotic fracture*[tiab] OR MOF[tiab] OR hip fracture*[tiab] OR spine fracture*[tiab] OR vertebral fracture*[tiab] OR humerus fracture*[tiab] OR humeral fracture*[tiab] OR wrist fracture*[tiab] OR forearm fracture*[tiab] OR radius fracture*[tiab]) Sort by: **Publication Date**	279,392	03:43:30

Embase

fragility fracture/ or hip fracture/ or spine fracture/ or humerus fracture/ or radius fracture/ or wrist fracture/ or (osteoporotic adj fracture*).ti,ab. or MOF.ti,ab. or (hip adj fracture*).ti,ab. or (spine adj fracture*).ti,ab. or (vertebral adj fracture*).ti,ab. or (humer* adj fracture*).ti,ab. or (wrist adj fracture*).ti,ab. or (forearm adj fracture*).ti,ab. or (radius adj fracture*).ti,ab.

AND

machine learning/ or artificial intelligence/ or deep learning/ or artificial neural network/ or support vector machine/ or (machine adj learning).ti,ab. or (transfer adj learning).ti,ab. or (deep adj learning).ti,ab. or (artificial adj intelligence).ti,ab. or (random adj forest*).ti,ab. or artificial neural network*.ti,ab. or ANN.ti,ab. or (support adj vector adj machine*).ti,ab. or SVM.ti,ab. or (gradient adj boosting*).ti,ab. or GBM.ti,ab. or Nomogram*.ti,ab. or XGBoost.ti,ab. or LightGBM.ti,ab. or (decision adj tree*).ti,ab. or LASSO*.ti,ab. or (backward adj selection).ti,ab. or (forward adj selection).ti,ab. or (stepwise adj selection).ti,ab. or (recursive adj elimination).ti,ab.

AND

predict* or prognostic* or (predictive adj model*).ti,ab.

AND

Artificial Intelligence Approaches for Osteoporotic Fracture Risk Prediction Using Administrative Health Data: A Systematic Review
Calcified Tissue International

Corresponding Author:
Benjamin Bakke Hansen
limit 4 to yr="2015 - 2026"

Supplementary Material B
Search date: 06-11-2025

Search History (5)

View Saved

# ▲	Searches	Results	Runtime	Type	Actions	Annotations
1	fragility fracture/ or hip fracture/ or spine fracture/ or humerus fracture/ or radius fracture/ or wrist fracture/ or (osteoporotic adj fracture*).ti,ab. or MOF.ti,ab. or (hip adj fracture*).ti,ab. or (spine adj fracture*).ti,ab. or (vertebral adj fracture*).ti,ab. or (humer* adj fracture*).ti,ab. or (wrist adj fracture*).ti,ab. or (forearm adj fracture*).ti,ab. or (radius adj fracture*).ti,ab.	159518	3.48	Advanced	Display Results More	
2	machine learning/ or artificial intelligence/ or deep learning/ or artificial neural network/ or support vector machine/ or (machine adj learning).ti,ab. or (transfer adj learning).ti,ab. or (deep adj learning).ti,ab. or (artificial adj intelligence).ti,ab. or (random adj forest*).ti,ab. or artificial neural network*.ti,ab. or ANN.ti,ab. or (support adj vector adj machine*).ti,ab. or SVM.ti,ab. or (gradient adj boosting*).ti,ab. or GBM.ti,ab. or Nomogram*.ti,ab. or XGBoost.ti,ab. or LightGBM.ti,ab. or (decision adj tree*).ti,ab. or LASSO*.ti,ab. or (backward adj selection).ti,ab. or (forward adj selection).ti,ab. or (stepwise adj selection).ti,ab. or (recursive adj elimination).ti,ab.	671694	6.23	Advanced	Display Results More	
3	(predict* or prognostic*).mp. or (predictive adj model*).ti,ab. [mp=title, abstract, heading word, drug trade name, original title, device manufacturer, drug manufacturer, device trade name, keyword heading word, floating subheading word, candidate term word]	3907602	5.32	Advanced	Display Results More	
4	1 and 2 and 3	1048	0.30	Advanced	Display Results More	
5	limit 4 to yr="2015 - 2026"	976	0.33	Advanced	Display Results More	

Save Remove Combine with: AND OR

Save All Edit Create RSS Create Auto-Alert View Saved Share Search History

Corresponding Author:
Benjamin Bakke Hansen

IEEE Xplore

"Osteoporotic Fractures" OR "Hip Fractures" OR "Spinal Fractures" OR "Humeral Fractures" OR "Radius Fractures" OR "Wrist Fractures" OR fracture* OR "osteoporotic fracture*" OR "major osteoporotic fracture*" OR MOF OR "hip fracture*" OR "spine fracture*" OR "vertebral fracture*" OR "humerus fracture*" OR "humeral fracture*" "wrist fracture*" OR "forearm fracture*" OR "radius fracture"

AND

"Machine Learning" OR "Artificial Intelligence" OR "Deep Learning" OR "Neural Networks, Computer" OR "Support Vector Machine" OR "machine learning" OR "transfer learning" OR "deep learning" OR "artificial intelligence" OR "random forest*" OR "artificial neural network*" OR ANN OR "support vector machine*" OR SVM OR "gradient boosting*" OR GBM OR Nomogram* OR XGBoost OR LightGBM OR "decision tree*" OR LASSO* OR "backward selection" OR "forward selection" OR "stepwise selection" OR "recursive elimination"

AND

predict* OR prognostic OR "predictive model*"

Showing **1-25** of **470** results for
("All Metadata":"Osteoporotic Fractures" OR "All Metadata":"Hip Fractures" OR "All Metadata":"Spinal Fractures" OR "All Metadata":"Humeral Fractures" OR "All Metadata":"Radius Fractures" OR "All Metadata":"Wrist Fractures" OR "All Metadata":fracture* OR "All Metadata":"osteoporotic fracture*" OR "All Metadata":"major osteoporotic fracture*" OR "All Metadata":MOF OR "All Metadata":"hip fracture*" OR "All Metadata":"spine fracture*" OR "All Metadata":"vertebral fracture*" OR "All Metadata":"humerus fracture*" OR "All Metadata":"humeral fracture*" "wrist fracture*" OR "All Metadata":"forearm fracture*" OR "All Metadata":"radius fracture") AND ("All Metadata":"Machine Learning" OR "All Metadata":"Artificial Intelligence" OR "All Metadata":"Deep Learning" OR "All Metadata":"Neural Networks, Computer" OR "All Metadata":"Support Vector Machine" OR "All Metadata":"machine learning" OR "All Metadata":"transfer learning" OR "All Metadata":"deep learning" OR "All Metadata":"artificial intelligence" OR "All Metadata":"random forest*" OR "All Metadata":"artificial neural network*" OR "All Metadata":ANN OR "All Metadata":"support vector machine*" OR "All Metadata":SVM OR "All Metadata":"gradient boosting*" OR "All Metadata":GBM OR "All Metadata":Nomogram* OR "All Metadata":XGBoost OR "All Metadata":LightGBM OR "All Metadata":"decision tree*" OR "All Metadata":LASSO* OR "All Metadata":"backward selection" OR "All Metadata":"forward selection" OR "All Metadata":"stepwise selection" OR "All Metadata":"recursive elimination") AND ("All Metadata":predict* OR "All Metadata":prognostic OR "All Metadata":"predictive model*") ×

☐ Conferences (367) ☐ Journals (88) ☐ Early Access Articles (8) ☐ Books (6)

☐ Standards (1)

Artificial Intelligence Approaches for Osteoporotic Fracture Risk Prediction Using Administrative Health Data: A Systematic Review
Calcified Tissue International

Corresponding Author:
Benjamin Bakke Hansen

Supplementary Material B
Search date: 06-11-2025

Web Of Science (Core Collection)

TS=("Osteoporotic Fractures" OR "Hip Fractures" OR "Spinal Fractures" OR "Humeral Fractures" OR "Radius Fractures" OR "Wrist Fractures" OR "osteoporotic fracture*" OR "major osteoporotic fracture*" OR MOF OR "hip fracture*" OR "spine fracture*" OR "vertebral fracture*" OR "humerus fracture*" OR "humeral fracture*" OR "wrist fracture*" OR "forearm fracture*" OR "radius fracture*")

AND

TS=("Machine Learning" OR "Artificial Intelligence" OR "Deep Learning" OR "Neural Networks, Computer" OR "Support Vector Machine" OR "machine learning" OR "transfer learning" OR "deep learning" OR "artificial intelligence" OR "random forest*" OR "artificial neural network*" OR ANN OR "support vector machine*" OR SVM OR "gradient boosting*" OR GBM OR Nomogram* OR XGBoost OR LightGBM OR "decision tree*" OR LASSO* OR "backward selection" OR "forward selection" OR "stepwise selection" OR "recursive elimination")

AND

TS=(predict* OR prognostic OR "predictive model*")

AND

Filters Applied: 2015-2026

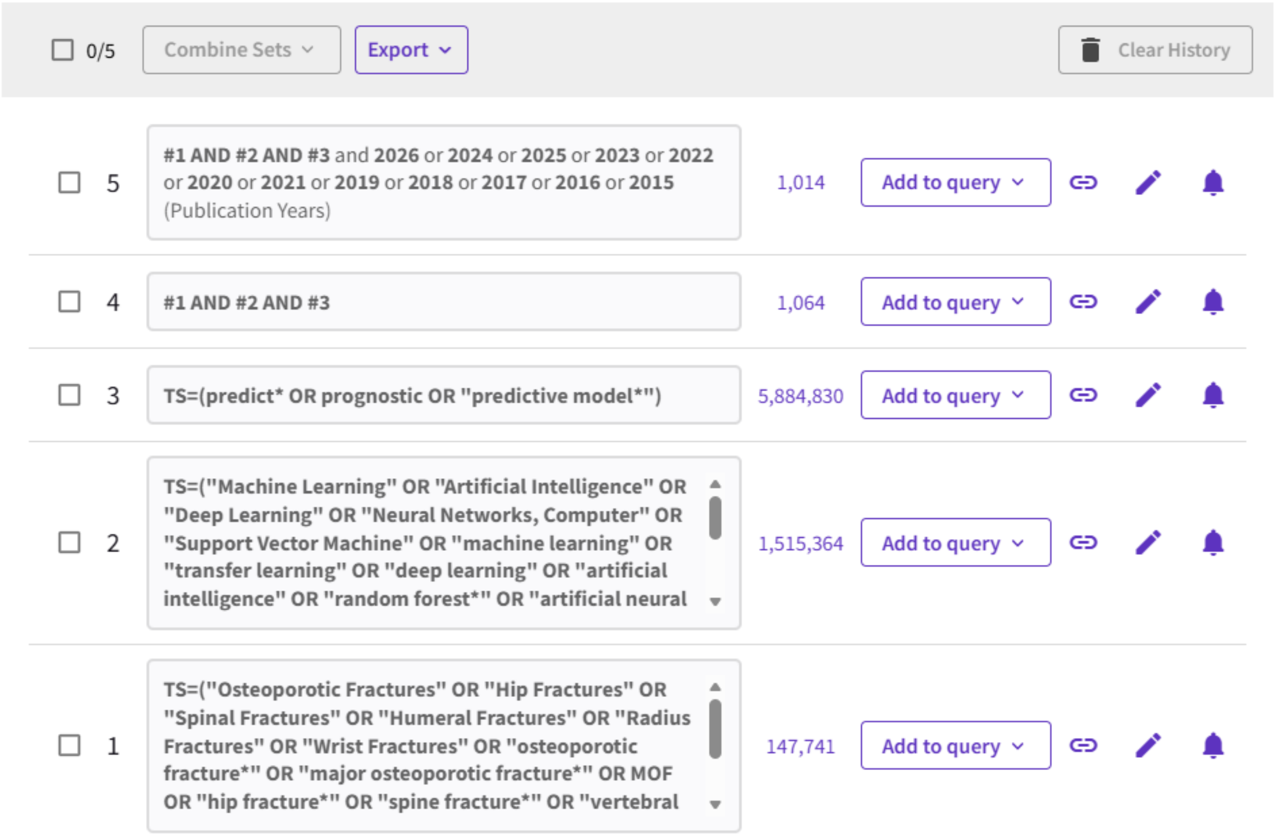

0/5 Combine Sets Export Clear History

#	Query	Results
5	#1 AND #2 AND #3 and 2026 or 2024 or 2025 or 2023 or 2022 or 2020 or 2021 or 2019 or 2018 or 2017 or 2016 or 2015 (Publication Years)	1,014
4	#1 AND #2 AND #3	1,064
3	TS=(predict* OR prognostic OR "predictive model*")	5,884,830
2	TS=("Machine Learning" OR "Artificial Intelligence" OR "Deep Learning" OR "Neural Networks, Computer" OR "Support Vector Machine" OR "machine learning" OR "transfer learning" OR "deep learning" OR "artificial intelligence" OR "random forest*" OR "artificial neural	1,515,364
1	TS=("Osteoporotic Fractures" OR "Hip Fractures" OR "Spinal Fractures" OR "Humeral Fractures" OR "Radius Fractures" OR "Wrist Fractures" OR "osteoporotic fracture*" OR "major osteoporotic fracture*" OR MOF OR "hip fracture*" OR "spine fracture*" OR "vertebral	147,741
