# Supplementary material for: Artificial Intelligence Approaches for Osteoporotic Fracture Risk Prediction Using Administrative Health Data: A Systematic Review
Source: Calcif Tissue Int. 2026 Jun 26;117(1):103. doi: 10.1007/s00223-026-01563-1 (PMC13303480; doi:10.1007/s00223-026-01563-1)
Supplement: Supplementary file 2 — Supplementary file2 (DOCX 26 KB) PROBAST Grading Rationales [file 223_2026_1563_MOESM2_ESM.docx]

**PROBAST Tool – Rationale behind each score in Supplementary Table S3**

*1. Almog et al. - Deep Learning With Electronic Health Records for Short-Term Fracture Risk Identification*

**Overall Judgment: Low ROB (+) | High concern regarding applicability (-)**

**ROB: Low Risk (+)**

**Participants (+):** The study used a very large US based EHR database (Optum). The cohort selection criteria were clearly defined and appropriate for the study’s aim.

**Predictors (+):** The model uses temporal sequences of ICD codes as predictors. This retrospective nature of the data precludes assessment bias.

**Outcome (+):** The fracture outcome was identified using a validated, rule-based algorithm applied to the Optum data.

**Analysis (+):** Uses a holdout set for validation, compares various models appropriately against baseline models and oversamples the training set, while correctly testing on the original, imbalanced data distribution.

**Applicability: High Concern (-)**

**Participants (-):** The data is from the US population, however the source as an EHR database and not a purely administrative database raises concerns regarding the generalizability as these data may differ from purely administrative data.

**Predictors (-):** Being developed on EHR data, it may contain more detailed and differently structured features than typical administrative data. The model’s core feature – learning from embedded precise temporal sequences of recorded diagnoses – may not be feasible or reliable in pure administrative databases, which are often aggregated for billing purposes or may lack necessary granularity in general.

**Outcome (+):** The fracture outcome was relevant and applicable.

*2. Reinold et al. - Potential of Health Insurance Claims Data to Predict Fractures in Older Adults*

**Overall Judgment: Low ROB (+) | Low concern regarding applicability (-)**

**ROB: Low risk (+)**

**Participants (+):** The study used a large claims database representing roughly 20% of the German population, providing a strong, unselected cohort. The exclusion of individuals with prior fractures was a clearly stated choice to focus on primary prevention and was applied consistently.

**Predictors (+):** Predictors from claims data were clearly defined and assessed retrospectively, eliminating assessment bias.

**Outcome (+):** Fractures were identified using ICD-10 codes, which is considered a highly valid method in German claims data.

**Analysis (+):** The methodological approach was sound, despite the finding of low to moderate performance.

**Applicability: High Concern (-)**

**Participants (+):** The cohort was derived from a large, generalizable administrative claims database.

**Predictors (-):** All predictors were sourced exclusively from claims data. The authors excluded patients with previous fractures, arguing that patients who had already experienced a fracture would have alerted caregivers and already taken measures [REF]. While this is a fair argument, we deem this as a reason for high concern of applicability in relation to the applicability of a generalistic model for predicting fracture risk. Prior fracture risk is a very strong predictor of current fracture risk, which Reinold et al. also mentions themselves in the paper [REF].

**Outcome (+):** The fracture outcome was relevant and applicable.

*3. Li et al. - Development and validation of sex-specific hip fracture prediction models using electronic health records*

**Overall Judgment: Low ROB (+) | High concern regarding applicability (-)**

**ROB: Low risk (+)**

**Participants (+):** The study used a large, representative EHR database from the public healthcare system in Hong Kong.

**Predictors (+):** Predictors included age and all diagnosis and drug prescription codes from the year prior the index date. These were clearly defined and retrospectively assessed.

**Outcome (+):** Hip fractures were identified using ICD-9 codes, which were previously validated in the database with a 100% PPV.

**Analysis (+):** The analysis is very strong, as it used not only an internal testing dataset but also validated the models on an independent and prospective cohort.

**Applicability: High Concern (-)**

**Participants (-):** While the model uses an independent population for validation, the model was developed on a Hong Kong population. This provides very valuable evidence for non-Caucasian populations, but it raises concerns about direct generalizability to other populations.

**Predictors (-):** Being developed on EHR data, it may contain more detailed and differently structured features than typical administrative data. It may not be feasible or applicable in pure administrative databases, which are often aggregated for billing purposes or may lack necessary granularity in general.

**Outcome (+):** The fracture outcome was relevant and applicable.

*4. Engels et al. - Osteoporotic hip fracture prediction from risk factors available in administrative claims data*

**Overall Judgment: High ROB (-) | High concern regarding applicability (-)**

**ROB: High risk (-)**

**Participants (+):** The study used a large administrative claims database. Exclusions were reasonable and clearly defined.

**Predictors (+):** Predictors were standard administrative data and were clearly defined.

**Outcome (+):** The outcome was identified using ICD-10 codes from hospital admissions.

**Analysis (-):** The authors applied random undersampling to the training set to balance the fracture and non-fracture classes. This method discards a vast amount of data from the majority class, leading to a loss of information and a high risk for the model to learn from biased, unrepresentative sample. The resulting poor calibration of the superlearner model in the validation set confirms that the model did not generalize well even within the same dataset.

**Applicability: High Concern (-)**

**Participants (-):** The data comes from the German agricultural sickness fund, which is limited to people working in agriculture and their families. This is quite a specific rural subpopulation and it is therefore of high concern how well a model developed on this group would generalize to a broader population.

**Predictors (+):** The model used only administrative claims data.

**Outcome (+):** The fracture outcome was relevant and applicable.

*5. Rietz et al. – Introducing FremML: A Decision-support Approach for Automated Identification of Individuals at High Imminent Fracture Risk*

**Overall Judgment: Low ROB (+) | Low concern regarding applicability (+)**

**ROB: Low risk (+)**

**Participants (+):** The study utilized high-quality and complete Danish national registry data. This is an ideal data source regarding administrative data. Exclusion criteria were appropriate and clearly defined.

**Predictors (+):** Predictors were extracted from the national registers and the data was retrospective eliminating the possibility of predictor assessment being influenced by the outcome.

**Outcome (+):** Both the primary outcome, Major Osteoporotic Fractures and Hip Fractures, was defined clearly and this method has previously been shown to be highly valid in Danish national health registers.

**Analysis (+):** The study was methodologically sound. The data was split appropriately (60/20/20), which is a standard practice. The use of a DART boosting algorithm with cross-validation and hyperparameter tuning is appropriate for developing high-performing ML models.

**Applicability: Low Concern (+)**

**Participants (+):** The model was developed on complete national administrative/register data.

**Predictors (+):** The model exclusively used predictors available from these administrative/register data.

**Outcome (+):** The fracture outcomes were relevant and applicable.

*6. Khalid et al. – Predicting Imminent Fractures in Patients With a Recent Fracture or Starting Oral Bisphosphonate Therapy: Development and International Validation of Prognostic Models*

**Overall Judgment: High ROB (-) | High concern regarding applicability (-)**

**ROB: High risk (-)**

**Participants (+):** The study utilized three large, representative databases: SIDIAP (Spain), CPRD (UK), and DHR (Denmark). The retrospective cohort design was appropriate, with clear inclusion criteria.

**Predictors (-):** Predictors were defined using standard administrative coding systems like ICD-10 and ATC-codes and harmonized across the three databases. The development set contained fracture numbers in the low hundreds depending on the outcome and sex. With 82 candidate variables the events per variable drops below 10 which violates the widely accepted heuristic by Peduzzi et al. (30).

**Outcome (+):** Outcomes (Hip, MOF and Any fracture) were pre-specified and identified using linked hospital and primary care records in the databases.

**Analysis (-):** The study accounted for overfitting by using LASSO regularization for variable selection in the development set. The authors performed both internal validation and double external validation and assessed calibration by intercept recalibration. However, the author’s did not report the fracture rates by sex in the external validation cohorts, despite developing and calibrating models stratified by sex. This raises concerns because we are unable to verify the the model’s recalibration and stability in the sex stratified models.

**Applicability: High Concern (-)**

**Participants (-):** The data sources used (SIDIAP, DHR, CPRD) are examples of large-scale administrative and routine clinical databases. The population of incident fracture and oral bisphosphonate users are highly relevant populations for fracture prediction models. However it is also a specific sub-category of individuals already in treatment uptake, which raises a concern for the generalizability of this population to the broader population of non-treated individuals.

**Predictors (+):** The model exclusively used predictors available from administrative/register data that was shared between the three databases.

**Outcome (+):** The fracture outcomes were relevant and applicable.

*7. Möller et al. – An enhanced Fracture Risk Evaluation Model (FREM) using national health data on morbidity and medications*

**Overall Judgment: Low ROB (+) | Low concern regarding applicability (+)**

**ROB: Low risk (+)**

**Participants (+):** The study utilized the entire Danish population aged 45 years or older, excluding those with prior osteoporosis diagnoses or treatment.

**Predictors (+):** Predictors were standard administrative data (ICD-10 codes and ATC-codes) using a 15-year lookback period.

**Outcome (+):** Both the primary outcome, Major Osteoporotic Fractures and the secondary Hip Fractures, was defined clearly and used a pre-validated algorithm for identifying incident fractures.

**Analysis (+):** The study was methodologically sound. The data was split appropriately (60/20/20), which is a standard practice. The study used Logistic Regression with LASSO regularization to feature select and prevent overfitting. They employed a three-way split of the data and used 10-fold-cross validation for hyperparameter tuning.

**Applicability: Low Concern (+)**

**Participants (+):** The cohort represents a general population suitable for opportunistic and automated screening because it uses nationwide register data.

**Predictors (+):** The model relies exclusively on standard ICD and ATC codes for the model feature spaces. These are almost universally available in administrative health and insurance databases, making the model feasibly transferable to other settings with similar coding systems.

**Outcome (+):** The fracture outcomes were relevant and applicable.
